# Are platelet volume indices of clinical use in COVID-19? A systematic review

**DOI:** 10.3389/fcvm.2022.1031092

**Published:** 2022-10-18

**Authors:** Sarah Daniels, Hua Wei, Martie van Tongeren, David W. Denning

**Affiliations:** ^1^Division of Population Health, Health Services Research and Primary Care, School of Health Sciences, University of Manchester, Manchester, United Kingdom; ^2^Manchester Academic Health Science Centre, University of Manchester, Manchester, United Kingdom; ^3^Division of Evolution, Infection and Genomics, School of Biological Sciences, University of Manchester, Manchester, United Kingdom

**Keywords:** COVID-19, mean platelet volume, platelet distribution width, platelet large cell ratio, thrombosis

## Abstract

**Background:**

The incidence of thrombotic complications is high in COVID-19 patients with severe disease. As key regulators of thrombus formation, platelets likely play a crucial role as mediators of severe acute respiratory syndrome coronavirus 2 associated pathogenesis. Studies have reported that parameters reflecting platelet size, known as platelet volume indices (PVI), are raised in patients with thrombosis and can predict poor outcomes. This systematic review evaluates the potential for PVI to be used as a predictor of COVID-19 morbidity and mortality.

**Methods:**

English and Chinese databases were searched electronically to identify studies reporting data on mean platelet volume, platelet distribution width or platelet-large cell ratio in COVID-19 patients. Included articles underwent a quality rating and descriptive narrative analysis.

**Results:**

Thirty-two studies were included in the systematic review. The results show a general trend for PVI to be raised in severe COVID-19 patients and non-survivors, with 14 studies reporting significant differences of baseline PVI between severe and mild disease. Nonetheless, longitudinal studies showed varying PVI trends over the course of the disease and evidence for PVI to be associated with disease progression was limited. The quality rating of 12 studies was poor, 16 were rated fair and four were good. Most studies were retrospective in design, used small study populations and did not consider confounding factors that influence platelet volume. Studies also contained technical flaws in PVI measurement, limiting the reliability of the results.

**Conclusion:**

The evidence on the clinical usefulness of PVI is greatly limited by the lack of prospective evaluation, together with technical problems in measuring PVI. Carefully designed prospective studies are warranted.

**Systematic review registration:**

https://www.crd.york.ac.uk/prospero/display_record.php?RecordID=304305, identifier CRD42022304305.

## Background

Coronavirus disease 2019 (COVID-19) disease is caused by severe acute respiratory syndrome coronavirus 2 (SARS-CoV-2). While most cases of COVID-19 are mild, some develop severe viral pneumonia with respiratory failure, that can result in death. Severe disease is predominantly observed in the elderly and those with underlying health conditions such as hypertension, diabetes and coronary heart disease (1). An unexpectedly high incidence of thrombosis has been reported (2, 3), and severity of COVID-19 disease is associated with elevated inflammatory markers and markers of coagulation such as D-dimer, fibrinogen and von Willebrand factor (1, 4). Moreover, COVID-19 autopsies have shown evidence of widespread microthrombosis in the lungs and other organs (5).

Circulating platelets play a central role in hemostasis and thrombosis, and platelets significantly contribute to immune responses during viral infection in a process termed “immunothrombosis” (6). Platelet hyperreactivity may contribute to immunothrombosis often seen in patients with COVID-19 (7). COVID-19 patients have higher levels of P-selectin expression in resting and activated platelets, elevated circulating platelet-leukocyte aggregates, increased aggregation, and thromboxane generation (8, 9). In addition, mild thrombocytopenia is observed in COVID-19 patients, and a progressive decline of platelet counts (PLT) was significantly associated with increased mortality (10). Moreover, pulmonary megakaryocytes are increased in COVID-19 patients with acute lung injury (11). Since the lung is considered an active site of megakaryopoiesis, a prothrombotic status leading to platelet activation, aggregation and consumption may trigger a compensatory pulmonary response (11).

Platelet activation markers are considered to be useful tools in evaluating risk factors of thrombosis in a variety of clinical conditions (12). While there are many methods used to test platelet activation for research purposes, most of the existing techniques are expensive, require trained personnel and take time to perform, limiting their use in clinical practice (12).

Circulation of larger, younger platelets reflect platelets activity and considered to be useful predictors of thrombotic events (13, 14). Platelet size can be assessed during a routine clinical blood test using automated hematology analyzers. Platelet volume indices (PVI) are a group of parameters that are routinely measured using automated hematology analyzers and include mean platelet volume (MPV), platelet distribution width (PDW) and platelet-large cell ratio (P-LCR). Their wide availability and low cost makes them appealing biomarkers for clinical research. Moreover, increased MPV is associated with thrombocytopenia, a hematological change often exhibited in COVID-19 patients (15). It has been proposed that megakaryocytes increase the production of large immature platelets as a compensatory mechanism for platelet consumption resulting from pulmonary microthrombi formation (16).

The aim of this systematic review is to evaluate the usefulness of PVI as clinical biomarkers for COVID-19 disease prognosis.

## Methods

### Search strategy

A review protocol was published on PROSPERO (ID: CRD42022304305). The review is reported in line with the Preferred Reporting Items for Systematic Reviews and Meta-Analyses (PRISMA) guidelines. We carried out a systematic search of the literature from Medline, Embase, PubMed, Web of Science, the Cochrane Central Register of Controlled Trials (CENTRAL) for all literature published up to 8th October 2021. Searches were limited to English language. Relevant studies were identified for all reported studies of associations between COVID-19 and platelet indices reflecting platelet size using the terms: “covid” OR “coronavirus” OR “ncov” OR “sars” OR “sars-cov” AND “mean platelet volume” OR “platelet distribution width” OR “platelet large cell ratio”. As an emerging research field a search of the preprint databases, MedRixv and BioRixv, was also conducted for all literature published from 1st January 2020 to 8th October 2021' using phrase terms for “mean platelet volume,” “platelet distribution width,” and “platelet large cell ratio.” The China Knowledge Resource Integrated (CNKI) database was searched for literature up to 18th October 2021, using the search terms “血小板(platelet)” AND “COVID-19”. Hand searching was also performed in the reference lists of relevant articles to identify additional eligible studies. See Supplementary material 1 for details of the search strategy.

### Inclusion/exclusion criteria

Studies were included in this review if they met the criteria as follows:

Inclusion criteria: (1) Adult patients with laboratory-confirmed COVID-19; (2) PVI biomarker (i.e., MPV, PDW and/or P-LCR); (3) investigation of an association between a PVI and disease severity and/or mortality in COVID-19; (4) blood tests performed at baseline for prognosis of severe disease or mortality [can be reported as 0–3 days of hospital or intensive care unit (ICU) admission or first test taken]; (5) we included only studies reporting PVI as continuous measures collected at baseline, comprising means or medians with measures of precision, e.g., standard deviation, standard error, confidence intervals or interquartile range (IQR); (6) original (experimental) research including randomized controlled trials, case-control studies, cohort studies, cross-sectional, case reports and series of cases; (7) articles in English or Chinese language.

Exclusion criteria: (1) under 18-year-olds (2) reviews, meta-analyses, conference abstracts, editorials, guidelines, commentaries, protocols (3) animal-based experiments; (4) *in vitro* studies; (5) unrelated studies; (6) studies focused on specific patient populations, e.g., diabetic or cancer patients; (7) no details of time of blood test; (8) no blood test taken at hospital or ICU admission; (9) articles with incomplete PVI data.

### Study selection

All records identified by the database search were screened by title and abstract. Chinese literature was translated into English by the Chinese speaking reviewer, HW. A random sample of 20% of the title/abstracts were screened from the literature and discussed between two authors (SD and HW), and the remaining abstracts were screened independently by SD. Studies considered relevant were evaluated in full text according to the prespecified inclusion and exclusion criteria. A random sample of 20% of the full text articles were screened and discussed between two authors (SD and HW), and the remaining full text articles were screened by SD.

### Data extraction

One reviewer (SD) extracted data from each study and compiled summary tables. A second reviewer (HW) randomly selected about 50% of the data extraction to check the accuracy. Any discrepancies identified were discussed and resolved between SD and HW and reflected in the remaining 50%. For all included studies, the following data was extracted: lead author, publication year, country, study design, study population (including age and % females), sample number, severity definition, day/time of blood test, subject exclusion if anti-platelet medication taken ≤ 10 days prior to test (yes or no), baseline PVI measurements and measures of effect.

### Quality assessment

All included articles were quality assessed using the National Institutes of Health's Quality Assessment Tools for Observational Cohort and Cross-Sectional Studies (17). The quality assessment was independently conducted by one reviewer (SD). A second reviewer (HW) randomly selected about 50% of the data extraction to check the accuracy. Any discrepancies identified were discussed and resolved between SD and HW and reflected in the remaining 50%. Each study was rated as poor, fair or good based on the details that were reported and consideration of the concepts for minimizing bias.

### Data synthesis and analysis

Given the heterogeneity of the clinical outcomes and PVI measurement, and the poor-fair quality of most studies (see section Results), we chose not to conduct a meta-analysis, and instead the study results are presented as a descriptive narrative analysis. Detailed evidence tables were created, and studies summarized by the reviewers. The technical limitations of the included studies are discussed.

## Results

### Study characteristics

**Figure 1:**
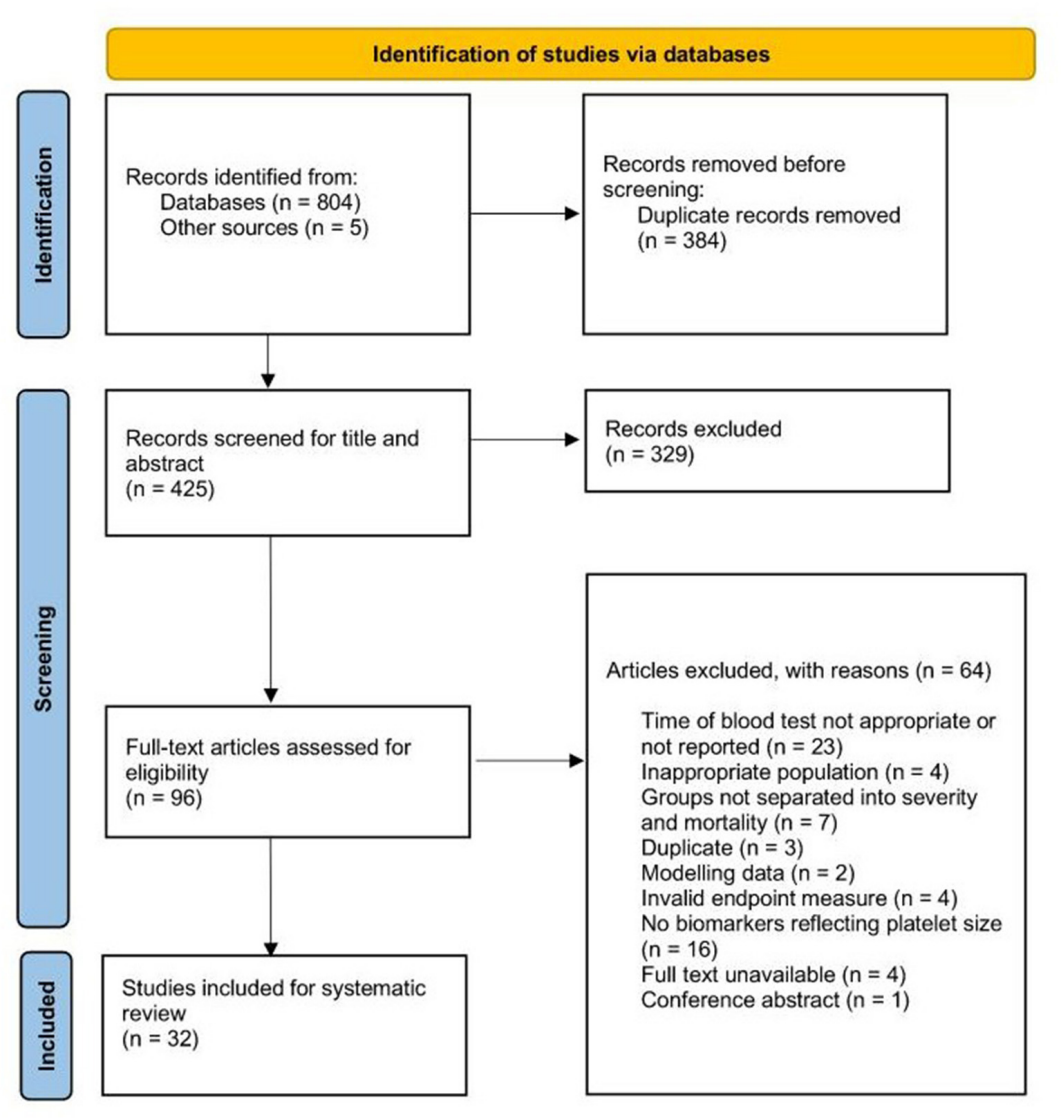
PRISMA diagram of search strategy.

We identified a total of 236 records from the OVID (Medline, Embase, and CENTRAL) search, 132 records from the Web of Science database, 133 records from PubMed, 196 records from the MedRxiv database, 107 from CNKI and five records from references searches. Of these, 329 were duplicates. Four-hundred and twenty-five records were title and abstract screened, and 96 were taken to full-text review. Sixty-four studies were excluded at the full-text review stage and 32 studies were included in the systematic review (Figure 1).

The baseline characteristics of the included studies are presented in Table 1. Fourteen studies were retrospective cohort, and five were prospective cohort studies. Six were cross-sectional and seven were longitudinal, observational studies. The largest number of studies were from Turkey (*n* = 10), followed by China (*n* = 8), and most were single-center studies (*n* = 27). Wu et al. (18) was an international multi-center study, but we extracted data for patients recruited from one center. Two studies were identified from preprint databases (19, 20) and three were published as letters (21–23). The disease outcomes for 21 studies were severity of COVID-19, and 12 studies assessed mortality. Two studies provided a comparison of patient groups combining severity and mortality (21, 24). Many studies classified disease severity as patients who were admitted to an ICU or presented with at least one of the clinical manifestations listed in national guidelines for severe or critical diagnosis of COVID-19 (19, 24–36). Only three studies reported subjects with similar ages between groups (*p* > 0.05) (30, 37, 38), and seven studies reported significantly different male:female ratios (*p* < 0.05) (18, 22, 37, 39–42). Only two excluded patients on antiplatelet drugs > 10 days prior to the blood test (31, 34) and another study adjusted for antiplatelet therapy in the data analysis (21). Values for platelet indices reported at hospital or ICU admission are listed in Tables 2, 3.

**Table 1:** Study characteristics.

**Study**					**Clinical outcome**		**Non-severe/** **survivor**			**Severe/** **non-survivor**			**Comparison of the two groups (** ***P*** **-value)**		**Quality rating**
**References**	**year**	**Country**	**Study design**	**Time of blood test**	**Non-severe/ survivor**	**Severe disease/ non-survivor**	**No**.	**Age**	**% female**	**No**.	**Age**	**% female**	**Age**	**% female**	
Ak et al. (25)	2021	Turkey	Retro., cohort, single center study	Admission	Non-severe according to findings evaluated during hospitalization	Severe survivors. Severe disease defined according to the national COVID-19 guidelines	380	61.15 ± 16.5	46.8	73	64.82 ± 12.1	41.1	< 0.001*	0.498*	Fair
					Severe survivors (defined as above)	Non-survivors (from the severe survivors group)	73	64.82 ± 12.1	41.1	22	49.52 ± 14.9	36.4	< 0.001*	0.498*	
Al-Nimer et al. (26)	2021	Iraq	Retro., cross., single center study	Admission	Recovered from illness and discharged	Non-survivors from the ICU	45	NR	NR	64	NR	NR	NR	NR	Fair
Alnor et al. (27)	2021	Denmark	Retro., cohort, dual center study	Admission	Non-severe	ICU admission and/or death	58	NR	NR	16	NR	NR	NR	NR	Fair
Asan et al. (28)	2021	Turkey	Retro., cohort, single center study	< 24 h of admission	Non-ICU admission	ICU admission according to listed criteria	668	41.0 [5.7]	52.7	27	69.0 [21.0]	44.4	< 0.001	NR	Fair
Barrett et al. (21)	2020	USA	Pros., cohort, single center study (letter)	< 24 h of admission	Hospitalized patients without thrombotic event or death	Hospitalized patients who had a thrombotic event or died	68	63.5 [48.5–73.0]	39.7	32	69.5 [63.0–80.0]	37.5	0.002	1.0	Poor
Barrett et al. (29)	2021	USA	Retro., long., single center study	0–28 days	Without critical illness	Critical illness defined as requiring mechanical ventilation or transfer to the ICU	NR^~^	NR	NR	NR^~^	NR	NR	NR	NR	Good
					Discharged	Death	NR	NR	NR	NR	NR	NR	NR	NR	
Bauer et al. (30)	2021	Germany	Pros., cohort, single center study	Admission	Non-ICU treatment	ICU admission	10	63.7 [52.5–71.0]	60	7	71.9 [57.5–76.8]	71	0.27	1.0	Fair
Comer et al. (31)	2021	Ireland	Retro., cohort, single center study	Day 0 and day 7 of hospital admission or transfer to ICU	Non-ICU admission	ICU admission	20	69.25 ± 17.7	35	34	59.4 ± 10.5	38	NR	NR	Poor
de la Rica et al. (38)	2020	Spain	Retro., cohort, single center study	< 24 h of admission	Non-ICU according to Son Latzer University Hospital for COVID-19 management	ICU according to Son Latzer University Hospital for COVID-19 management	27	66.30 ± 14.90	33	21	65.57 ± 12.87	33	0.856	1	Fair
Ding et al. (43)	2020	China	Retro., cohort, single center study	Admission	Non-severe defined according to the coronavirus pneumonia diagnosis and treatment program, and the criteria of clinical classification	Severe defined according to the coronavirus pneumonia diagnosis and treatment program, and the criteria of clinical classification	57	46 [35–60]	57.9	15	67 [55–76]	40	NR	NR	Poor
Dogan et al. (32)	2021	Turkey	Retro., cohort,single center study	Admission	Non-ICU defined according to the Turkish Ministry of Health COVID-19 guidelines	ICU defined according to the Turkish Ministry of Health COVID-19 guidelines	131	NR	NR	20	NR	NR	NR	NR	Fair
Eraybar et al. (41)	2021	Turkey	Retro., cohort, single center study	First admission	Survivor	Non-survivor defined as 28-day mortality	905	NR	53.7	33	NR	36.4	NR	0.05	Poor
Giusti et al. (23)	2020	Italy	Retro., cohort, single center study (letter)	Admission	Discharged	Non-discharged	117	59.9 ± 14.0	36.8	92	73.6 ± 11.6	35.9	< 0.001	0.895	Poor
					Survivor	Non-survivor	178	63.5 ± 14.0	37.1	31	63.5 ± 14.0	32.3	< 0.001	0.607	
Guclu et al. (44)	2020	Turkey	Retro.,cohort, single center study	Day of admission	Patients with room air oxygen saturation ≥90%	Patients with room air oxygen saturation < 90%	81	56.52 ± 15.95	45.7	134	69.04 ± 14.26	43.3	< 0.001	0.732	Fair
					Survivor	Non-survivor	159	61.15 ± 16	45.3	56	73.34 ± 12.58	41.1	< 0.001	0.697	
Higuera-De-La-Tijera et al. (33)	2021	Mexico	Pros., cohort, single center study	Admission	Patients with COVID-19 pneumonia without severity criteria for ICU admission (evaluated by intensive care medical staff)	ICU admission (evaluated by intensive care medical staff)	139	49.1 ± 12.8	32.4	27	58.6 ± 12.7	25.9	0.001	0.51	Fair
Incir et al. (34)	2021	Turkey	Pros., cohort, single center study	First day of admission	Non-severe defined as patients not requiring oxygen support	Severe defined as the mechanical ventilation requirement, admission to the intensive care unit (ICU), and death	110	45 [18–89]^Ψ^	50	44	63 [20–91] ^Ψ^	17	0.003	0.607	Good
Jamshidi et al. (20)	2021	Iran	Retro., cohort,multi-center study	1–3 days of ICU admission	Survivor	Non-survivor	105	58.0 [47.0–73.0]	46.7	158	72.5 [64.0–80.75]	53.3	< 0.001	NR	Poor
Karaasla-n et al. (45)	2021	Turkey	Retro., cohort, multi center study	Admission	Survivor	Non-survivor	182	50.6 ± 15.4	50	24	69.7 ± 16.0	50	< 0.001	NR	Poor
Kilercik et al. (24)	2021	Turkey	Retro., long., single center study	0–30 days	Non-critical and critical survivors. Followed the COVID-19 directory of the Ministry of Health of the Republic of Turkey for listed criteria.	Critical non-survivors. Followed the COVID-19 directory of the Ministry of Health of the Republic of Turkey for listed criteria.	82	49.2 ± 15.1 and 58.6 ± 16.4	37.8	15	69.2 ± 10.6	20	< 0.001*	NR	Good
Ko et al. (39)	2020	China	Retro., cross., multi center study	< 24 h of admission	Survivor	Non-survivor	195	50.39 ± 15.00	52.8	212	68.90 ± 11.93	38.3	< 0.001^$^	< 0.001^#^	Poor
Lanini et al. (46)	2020	Italy	Pros., long., single center study	0–21 days	Survivor. Patients who recovered and were discharged from hospital or who were still hospitalized within 30 days after symptoms onset	Non-survivor. Died within 30 days after onset of symptoms.	338	< 60 yrs 95.73% >60 yrs 84.19%	87.7	41	< 60 yrs 4.27% >60 yrs 15.81%	12.3	NR	NR	Fair
Mao et al. (35)	2021	China	Retro., long., single center study	0–>25 days	Moderate disease according to the Chinese management guidelines for COVID-19 (7th edition) released by the NHCC	Severe or critically ill according to the Chinese management guidelines for COVID-19 (7th edition) released by the NHCC	82	62 (53–68)	45	45	68 (61–73)	23	0.003	0.684	Fair
Mertoglu et al. (47)	2021	Turkey	Retro., long., single center study	Day of admission	Non-ICU admission	ICU admission	532	47.5 [32–64.75]	42.5	23	59.0 [41.0–75.0]	43.5	0.011	0.925	Fair
Ouyang et al. (48)	2020	China	Retro., long., single center study	First test after incharge	Survivor COVID-19 patients discharged, transferred for further treatment or due to the presence of improved symptoms or the closure of this emergency hospital	Non-survivor. COVID-19 patients who died by 18 March 2020	82	55.7^ϕ^	NR	25	63.5^ϕ^	NR	0.018	NR	Fair
Rolla et al. (22)	2021	Italy	Pros., cohort, single center study (letter)	Admission	Survivor	Non-survivor	152	62 [51–73]	43	31	80 [74–85]	19	< 0.001	0.022	Poor
San et al. (49)	2021	Turkey	Cross.; single center study	Admission	Non-severe based on clinical manifestation	Severe based on a slightly modified interim guidance of the World Health Organization was used.	344	42 ± 23	44.2	44	67.5 ± 13.75	38.6	< 0.0001	0.59	Fair
Song et al. (19)	2020	China	Retro., cross., single center study	First inhospital results	Mild and moderate disease based on the New Coronavirus Pneumonia Prevention and Control Program (6th edition) by NHCC	Severe and critical disease based on the New Coronavirus Pneumonia Prevention and Control Program (6th edition) by NHCC	31	48.0 [37.0–59.0]	48.4	42	55.5 [48.0–64.3]	28.6	0.039	0.083	Poor
Tsui et al. (40)	2020	China	Retro., cross., multi center study	At admission and day 5	Satisfactory: progressing well and likely to be discharged soon; stable: with mild ILI symptoms;	Critical: require intubation, or ECMO or in shock; (2) serious: require oxygen supplement of 3 L or more per minute	502 and 485	35.6 ± 16.8 and 37.6 ± 17.5	41.4 and 52.2	50	60.6 ± 14.0	36	< 0.0001*	0.0011*	Poor
Waris et al. (36)	2021	Pakistan	Retro., cross., single center study	2nd day of admission	Mild and moderate disease according to the NHCC guidelines	Severe and critical disease according to the NHCC guidelines	76	43.24 and 49.1	30.2	25	56.6 and 62.1	32	NR	NR	Fair
Wu et al. (18)	2020	China	Retro., cohort, single center study	Obtained from medical records when the patients were admitted for the first time	If none of the “severe disease” criteria were met during the whole hospitalization process	Severe disease if one of the listed criteria were met, including respiratory distress or failure, ICU admission or death	217	42.0 [33.0–59.0]	58.5	82	62.0 [53.0–71.8]	42.7	< 0.001	0.019	Poor
Xiong et al. (42)	2020	China	Retro., long., single center study	Day 1–14	Mild/non-severe. Clinical classification at admission based on APACHE II, SOFA and CURB-65 criteria	Severe and critical. Clinical classification at admission based on APACHE II, SOFA and CURB-65 criteria	355	47 [34–61] and	65	166 and 139	62 [52–70] And 67 [54–76]	53 and 33	< 0.001*	< 0.001*	Fair
Zhong and Peng (37)	2021	China	Retro., cohort, dual center study	Admission	MPR < 7.44	MPR > 7.44 Severe pneumonia of COVID-19 defined by clinical manifestation	59	41.0 [34.0-58.0]	57.6	26	51.0 [35.5–68.3]	26.9	0.064	0.009	Good

^$^calculated from the raw data using a two-tailed Welch's unpaired test. ^#^calculated from the raw data using a two-tailed Fisher's exact test. *Compares three patient groups. Data presented as mean ± standard deviation, median [range] (marked with a ^Ψ^), average (marked with a ϕ) or median [interquartile range]. ^~^Study reported a total cohort of 3,915 COVID-19 patients. APACHE, Acute Physiology and Chronic Health Evaluation; Cross., Cross-sectional study; ECMO, extracorporeal membrane oxygenation; ICU, Intensive care unit; ILI, influenza-like illness; long., longitudinal; MPR, Mean platelet volume/platelet count ratio; NHCC, National Health Commission of China; NR, not reported; Pros., prospective; Retro., Retrospective; SOFA, Sequential Organ Failure Assessment.

**Table 2:** Values of platelet indices in the COVID-19 patient severe and non-severe groups at admission.

**First author**	**Year**	**Platelet indices**	**Non-severe patients**	**Severe patients**	**Comparison of the two groups (*p*-value)**
Ak et al. (25)	2021	PLT	203 ± 77.52	179 ± 81.46	NR
		MPV	9.6 ± 1.08	10 ± 1.16	NR
Alnor et al. (27)	2021	PLT	188 [161–271]	174 [127–203]	**0.037**
		MPV	10.5 [9.6–10.9]	10.9 [10.1–11.8]	0.126
		PDW	12.1 [10.5–13.2]	13.0 [11.6–14.5]	0.097
		P-LCR	28.5 [22.9–32.7]	31.1 [25.9–39.4]	0.093
Asan et al. (28)	2021	PLT	221 ± 60	198 ± 61	0.081
		MPV	9.9 ± 4.2	10.1 ± 1.6	0.241
		MPR	0.044 ± 0.018	0.053 ± 0.028	**0.049**
Bauer et al. (30)	2021	PLT	187.0 [161.0–233.0]	208.0 [167.0–233.0]	1.0
		MPV	10.2 [9.9–10.6]	10.5 [10.1–10.6]	0.49
Comer et al. (31)	2021	PLT	242 [105–488]^ω^	213 [40–550] ^ω^	0.6
		MPV	9.8 [8.7–13] ^ω^	10.8 [9–13] ^ω^	**0.015**
de la Rica et al. (38)	2020	PLT	228.02 ± 108.82	209.56 ± 77.97	0.678
		MPV	7.92 ± 1.25	8.18 ± 1.19	0.596
		PDW	16.81 ± 1.24	16.99 ± 0.97	0.333
Ding et al. (43)	2020	PLT	180 [149–227]	160 [134–216]	0.515
		MPV	9.1 [7.1–10.0]	9.7 [9.2–11.6]	**0.017**
Dogan et al. (32)	2021	PLT	234 ± 74	235 ± 94	0.933
		MPV	10.30 [9.70–10.90]	11.05 [10.35–11.75]	**0.0042**
Giusti et al. (23)	2020	PLT	191 [156–235]	183 [141–217]	0.088
		MPV	10.4 [9.9–11.1]	10.9 [10.5–11.9]	**0.001**
Guclu et al. (44)	2020	PLT	187.4 ± 59.82	208.63 ± 135.72	0.573
		MPV	9.18 ± 1.24	9.61 ± 1.76	0.129
		PDW	17.37 ± 2.32	17.72 ±2.52	0.142
Higuera-De-La-Tijera et al. (33)	2021	PLT	226.4 ± 86.2	219.7 ± 73.1	0.77
		MPV	8.4 ± 0.9	8.9 ± 0.9	0.11
Incir et al. (34)	2021	PLT	244 [155–460] ^ω^	196 [150–636] ^ω^	0.065
		MPV	10.8 ± 1.10	12.1 ± 1.22	**< 0.001**
		PDW	12.4 [7.50–24.5] ^ω^	15.6 [10.3–21.9] ^ω^	**0.001**
		P-LCR	31.4 ± 8.84	40.5 ± 9.30	**0.001**
Mertoglu et al. (47)	2021	PLT	233.0 [193.0–278.0]	219.0 [176.0–312.0]	0.872
		MPV	10.1 [9.5–10.7]	10.8 [9.6–11.7]	0.052
		P-LCR	25.60 [21.0–30.61]	32.70 [22.8–38.0]	**0.033**
San et al. (49)	2021	PLT	210 ± 96	199.5 ± 127	0.95
		MPV	8.2 ± 1.2	8.6 ± 1.75	**0.003**
		MPR	0.04 ± 0.02	0.04 ± 0.03	0.453
		LMR	6.42 ± 4.01	8.84 ± 5.5	**< 0.0001**
Song et al. (19)	2020	PLT	178.0 [127.0–239.0]	189.0 [154.0–231.0]	0.321
		PDW	12.8 [10.7–13.9]	12.3 [11.0–13.5]	0.643
Tsui et al. (40)	2020	PLT	Stable: 0.60 ± 0.19	Critical: 0.54 ± 0.19	**0.0094** *
			Satisfactory: 0.58 ± 0.20		
		MPV	Stable: 0.88 ± 0.12	Critical: 0.88 ± 0.10	0.9139
			Satisfactory: 0.87 ± 0.15		
Waris et al. (36)	2021	PLT	Mild: 217.03 [191.52–240.55]	Severe: 205.55 [120.38–290.72]	0.16*
			Moderate: 223.73 [185.93–261.54]	Critical: 165.06 [123.44–206.67]	
		MPV	Mild: 9.02 [8.60–9.43]	Severe: 9.34 [8.62–10.6]	0.31*
			Moderate: 12.60 [5.93–19.26]	Critical: 9.74 [9.15–10.33]	
		PDW	Mild: 12.18 [11.81–12.56]	Severe: 12.53 [11.31–13.75]	0.31*
			Moderate: 13.70 [10.92–16.48]	Critical: 12.21 [12.05–14.37]	
		P-LCR	Mild: 23.08 [21.28–24.87]	Severe: 23.52 [18.36–28.68]	0.39*
			Moderate: 25.40 [21.12–29.68]	Critical: 26.49 [22.29–30.69]	
Wu et al. (18)	2020	PLT	174.0 [140.8–214.5]	149.0 [116.5–188.8]	**< 0.001**
		MPV	9.8 [9.2–10.6]	10.2 [9.4–10.09]	0.016
		PDW	12.9 [10.7–16.3]	12.4 [10.8–15.8]	0.371
		P-LCR	24.0 [19.7–30.1]	26.8 [21.1–32.3]	0.028
Xiong et al. (42)	2020	PLT	Mild and non-severe: 187.0 [153.0–235.0]	Severe: 193.0 [154.0–248.0]	< 0.001*
				Critical: 122.0 [91.0–168.0]	
		MPV	Mild and non-severe: 9.70 [9.00–10.40]	Severe: 9.60 [8.93–10.30]	< 0.001*
				Critical: 10.15 [9.30–11.0]	
		PDW	Mild and non-severe: 15.70 [10.80–16.33]	Severe: 15.90 [10.62–16.50]	0.9*
				Critical: 13.40 [10.70–16.40]	
		P-LCR	Mild and non-severe: 23.30 [18.93–29.20]	Severe: 22.95 [18.22–28.78]	< 0.001*
				Critical: 26.20 [21.08–34.30]	

Data presented as mean ± standard deviation, median [min-max range] (marked with a ω) or median [interquartile range]. Units for platelet indices are PLT: 10^9^/L; MPV: fL; PDW: %; P = LCR: %. LMR, lymphocyte/mean platelet volume ratio; MPR, mean platelet volume/platelet count ratio; MPV, mean platelet volume; NS, not significant; P-LCR, platelet-large cell ratio; PLT, platelet count; PDW, platelet distribution width. ^*^Comparison of three or more patient groups. Significant *p*-values (<0.05) in bold.

**Table 3:** Values of platelet indices in the COVID-19 patient survivor and non-survivor groups at admission.

**First author**	**Year**	**Platelet indices**	**Survivors**	**Non-survivors**	**Comparison of the two groups (*p*-value)**
Ak et al. (25)	2021	PLT	179 ± 81.46	191.5 ± 100.3	NR
		MPV	10 ± 1.16	9.4 ± 1.64	NR
Al-Nimer et al. (26)	2021	MPV	8.9 ± 0.1	9.1 ± 0.1	0.310
		PDW	41.6 ± 1.2	41.4 ± 1.2	0.921
Barrett et al. (21)^ω^	2020	PLT	205.0 [164.8–253.8]	187.5 [147.5–257.5]	0.385
		MPV	10.55 [10.1–11.2]	11.00 [10.5–11.9]	**0.022**
Eraybar et al. (41)	2021	MPV	9.60 [9.00–10.30]	9.70 [9.20–11.10]	>0.05
		LMR	0.21 [0.15–0.29]	0.12 [0.07–0.20]	**< 0.001**
Giusti et al. (23)	2020	PLT	189 [149–233]	174 [111–204]	0.127
		MPV	10.5 [10.0–11.3]	11.4 [10.6–12.4]	**< 0.001**
Guclu et al. (44)	2020	PLT	207.69 ± 123.06	180.59 ± 78.14	0.094
		MPV	9.34 ± 1.37	9.77 ± 2.11	0.189
		PDW	17.44 ± 2.35	18.02 ±2.69	**0.040**
Jamshidi et al. (20)	2021	PLT	196.0 [151.5–260.0]	179.0 [125.0–255.0]	**0.04**
		MPV	9.7 [9.175–10.5]	10.0 [9.3–10.7]	0.3
		PDW	12.8 [11.5–14.0]	13.2 [11.4–14.7]	0.32
		P-LCR	24.4 [19.85–29.3]	26.7 [21.05–30.825]	0.07
Karaaslan et al. (45)	2021	PLT	199.93 ± 79.6	199.42 ± 124.9	0.97
		MPV	9.60 ± 1.4	9.72 ± 1.0	0.57
Ko et al. (39)	2020	PLT	212.48 ± 82.00	159.45 ± 86.54	< 0.0001*
		MPV	10.63 ± 0.87	11.16 ± 0.94	< 0.0001*
		PDW	12.29 ± 2.02	13.59 ± 2.64	< 0.0001*
		P-LCR	29.60 ± 7.08	33.88 ± 7.42	< 0.0001*
Lanini et al. (46)	2020	PLT	n/a	n/a	**0.019** ^**%**^
		MPV	n/a	n/a	**0.001** ^**%**^
Ouyang et al. (48)	2020	PLT	214.66 ± 91.61	178.77 ± 93.70	0.1
		MPV	9.23 ± 1.05	10.01 ± 1.15	**0.003**
		PDW	16.18 ± 0.42	16.63 ± 0.49	**< 0.001**
		P-LCR	21.60 ± 7.00	26.75 ± 7.69	**0.003**
Rolla et al. (22)	2021	PLT	186.50 [152.25–226.50]	148.00 [125.00–197.00]	**0.005**
		MPV	10.50 [9.90–11.00]	11.40 [10.70–12.10]	**< 0.001**
		P-LCR	28.5 [23.35–33.20]	35.90 [31.20–42.40]	**< 0.001**

Data presented as mean ± standard deviation or median [interquartile range]. Units for platelet indices are PLT: 10^9^/L; MPV: fL; PDW: %; *P* = LCR: %. LMR, Lymphocyte/mean platelet volume ratio; MPV, mean platelet volume; P-LCR, platelet-large cell ratio; PLT, platelet count; PDW, platelet distribution width. ^*^Author calculated from raw data using Welch's unpaired *t*-test for continuous data. ^ω^Study outcomes were death and/or thrombosis event. Significant *p*-values (<0.05) in bold. ^%^Temporal difference between the patient groups.

Although there was a trend toward lower PLT in the severe and non-survivor groups for most studies, it was reported to be significantly lower in five studies of severe patients vs. mild disease (18, 27, 31, 40, 42) and four studies of non-survivors vs. survivors (20, 22, 24, 39). A longitudinal study reported a higher PLT in non-survivors compared to survivors at the early stages of the disease, while the opposite was evident at the end of the follow-up (46). Only one study reported a mean PLT for severe COVID-19 patients that was within the mild thrombocytopenia range (100–150 × 10^9^) (18). In non-survivors, Rolla et al. (22) reported mild thrombocytopenia and Kilercik et al. (24) reported moderate thrombocytopenia (50–99 × 10^9^).

### Quality assessment

Twelve studies were rated poor, 16 were rated fair and four were good (Supplementary material 2). Most studies clearly specified the location, study time period and demographics of the selected participants. Nonetheless, there are several reasons for rating the studies based on potential bias. Most studies were retrospective in design. Consequently, most researchers had no control over the exposure assessment, therefore many did not fully describe the methods used to measure the platelet parameters. Fourteen studies provided the number of eligible patients and the total number included in the study (25, 27, 28, 33–35, 37–42, 45, 46), of which three had a participation rate < 50% (33, 41, 45). Possible bias related to participation rate could not be ascertained in the remaining 18 studies. Moreover, only two studies (26, 33) included a justification for sample size, hence, most authors were unable to make a valid inference about the population being studied.

Pre-analytical and analytical variables, such as the anticoagulant used, the time between blood collection, storage temperature and instrument type are known to significantly affect MPV measurements (50). For example, platelets collected into ethylenediaminetetraacetic acid (EDTA) anticoagulant undergo time-dependent platelet swelling and activation. Only eight (25%) of included studies reported the anticoagulant used (EDTA for all of them) (21, 24, 26, 27, 30, 34, 36, 37) and four (13%) reported the time to analysis (24, 28, 34, 37). A larger proportion of studies (41%) reported the analyzer used to measure PVI (21, 22, 24, 27–30, 32, 34, 36, 37, 43, 47), with the Sysmex XN series reported most frequently.

PVI may be influenced by various demographic factors including age, although no conclusive data are available on this topic (51). Of the 32 included studies, 12 considered age as a confounding variable and adjusted statistically for its impact on the relationship between PVI and disease severity or mortality (21, 23, 24, 28, 29, 33, 37, 38, 42, 44, 46, 47).

### PVI as biomarkers of severity in COVID-19 patients

#### Baseline PVI measurements

Nineteen studies compared the baseline levels of MPV in COVID-19 patients at hospital admission (18, 21, 23, 25, 27, 28, 30–36, 38, 40, 42–44, 47, 49), of which nine reported a significantly higher MPV in the severe COVID-19 patients compared with non-severe patients (18, 21, 23, 31, 32, 34, 42, 43, 49). Of nine studies that assessed PDW in COVID-19 patients (18, 19, 27, 34–36, 38, 42, 44), one study reported a significantly higher PDW in the severe group compared to the non-severe group at admission (34). Of seven studies assessing P-LCR in COVID-19 patients (18, 27, 34–36, 42, 47), three reported significantly higher P-LCR in the severe patient group at admission (34, 42, 47).

Two studies measured PVI on different days of hospital admission. A retrospective cohort study (44) reported a non-significant mean MPV at day 0 in COVID-19 patients with room air oxygen saturation < 90%, but significantly higher MPV at day 3, though this significance was marginal (*p* = 0.043). No significantly different PDW was observed for both days. Comer et al. (31) observed higher median MPV in ICU patients compared with non-ICU patients at day 7 vs. day 0 (*p* = 0.0014 and *p* = 0.015, respectively). It should be noted, however, that this difference was due to the MPV decreasing in the non-ICU patients at day 7, rather than the MPV increasing in the ICU patients.

#### Longitudinal analysis

Three retrospective longitudinal studies evaluating PVI in non-severe and severe COVID-19 patients reported conflicting results. A Chinese study of non-severe/mild, moderate and critical patients (42), identified significantly higher median MPV and P-LCR in the more severe groups (*p* ≤ 0.001) from day 1 to day 14 of hospitalization. PDW significantly increased from day 3 onwards. In contrast, a Turkish study (47) observed higher median MPV and P-LCR in ICU patients compared to non-ICU patients except > 7–8 days. Severe or critically ill COVID-19 patients without existing hematologic disease, were reported to have significantly higher mean MPV and P-LCR at >25 days after hospitalization (*p* ≤ 0.01) compared to moderate patients, with no significant difference prior to this (35). However, they reported no significant difference between patient groups for PDW for the entire course of the disease.

#### Measures of association

After adjustment for variables including age, comorbidities and prior medication, Barrett et al. (29) reported that patients with the highest MPV tertile at baseline had higher odds of requiring mechanical ventilation or transfer to the ICU (OR 1.5, 95% CI 1.3–1.8). Importantly, they show that this association remains significant in patients without thrombocytopenia, demonstrating that the high MPV was independent of low PLT. A prospective cohort study of 14 patients who experienced a thrombotic event, reported that MPV was not associated with thrombosis after adjustment for multiple variables including age, sex, anti-platelet therapy and PLT (21). Multivariate logistic regression to assess the effect of age and gender, found that P-LCR was not significant in determining admission to the ICU (47). Likewise, univariant logistic regression identified no association with MPV and ICU requirement in COVID-19 patients (32).

#### Other PVI

Zhong and Peng (37) investigated the relationship between mean platelet volume/platelet count ratio (MPR) and the prognosis of COVID-19. They found that high baseline MPR (>7.44 fL) was significantly associated with severe pneumonia in COVID-19 patients. Contrary to this, MPR was not found to be a significant hematological marker for severity in a cross-sectional study (49) and retrospective cohort study (28). San et al. reported a significantly higher median lymphocyte/mean platelet volume ratio (LMR) in severe patients (*p* ≤ 0.0001) (49), though multivariate logistic regression revealed that it was not an independent risk factor for severe disease.

### PVI as biomarkers of mortality in COVID-19 patients

#### Baseline PVI measurements

Eleven studies assessed baseline MPV in COVID-19 patients who survived and died (20–23, 25, 26, 39, 41, 44, 45, 48). Of which, six studies reported a significantly higher MPV in non-survivors at hospital admission (21–23, 39, 44, 48). Five studies assessed baseline PDW in COVID-19 mortality (20, 26, 39, 44, 48), and three reported higher PDW in non-survivors at admission (39, 44, 48). P-LCR at hospital admission was assessed in only four included studies (20, 22, 39, 48), and three reported significantly higher P-LCR in non-survivors (22, 39, 48).

Guclu et al. (44) reported that deceased COVID-19 patients had non-significant MPV when compared to survivors on the first day of hospital admission (*p* = 0.005), but significantly higher MPV on the third day. Conversely, they found significantly higher PDW in the non-survivor group at both admission and the third day (day 0: *p* = 0.040; day 3: *p* = 0.006).

#### Longitudinal analysis

Varying trends were observed for the longitudinal studies. A retrospective study of 98 patients identified a significantly higher MPV, PDW and P-LCR in non-survivors for the first test of the hospital stay (48). MPV and P-LCR increased over the course of the disease, with non-survivors showing an average MPV that was above the normal reference range for the last test. In contrast, the temporal trends analysis appears to show PDW decreasing in the survivors over time.

A comprehensive study of 379 COVID-19 patients over 21 days reported patterns of temporal variations for MPV (46). Tests showed significantly higher MPV in survivors compared with non-survivors at the beginning of the disease (*p* < 0.001). While the opposite was observed at the end of the follow-up, with MPV significantly higher in non-survivors than in survivors (*p* < 0.001). Over time, MPV tended to normalize in survivors and steadily increase in non-survivors, exceeding the upper normal limit value (11 fL) by the seventh day after symptoms' onset. Paradoxically, age was not significantly associated with higher MPV, which contrasted with other included studies.

Lastly, a retrospective study that observed 97 patients over 30 days of hospitalization (24), reported a significantly higher MPV, PDW and P-LCR in critical non-survivors compared with non-critical and critical survivors (*p* = 0.014, *p* = 0.011, and *p* = 0.006, respectively). As the disease progressed, all PVI gradually increased, with the differences among the groups remaining significant over time (*p* < 0.05). Moreover, trends analysis of MPV showed a distinctive divergence for all three groups after 10 days.

#### Measures of association

A prospective cohort study of 183 COVID-19 patients reported that MPV and P-LCR were shown not to be independent predictors of in-hospital mortality in multivariate analysis (22). In contrast, a prospective cohort study of 100 patients, reported MPV was significantly associated with all-cause mortality after adjustment for multiple variables such as age, sex, anti-platelet therapy and PLT (OR 2.33, 95% CI 1.27–4.67) (21). Guclu et al. showed that an increase of 1-unit MPV difference (between 1st and 3rd day), significantly increases the probability of death within 28 days (OR 1.762, 95% CI 1.272–2.440) (44).

## Discussion

For patients with severe COVID-19 infection, early decision making is critical for successful clinical management to prevent the development of acute respiratory distress syndrome and possible death. To the best of our knowledge, this is the first comprehensive systematic review to evaluate the usefulness of PVI as early predictors for severity and mortality in COVID-19 patients based on the current literature.

The quality assessment demonstrated that currently most studies published in this field are poor, fair at best. As such, we decided that the premises of meta-analysis were not met, and that the data used to generate the composite outcome would not be reliable. For this reason, we chose to perform a narrative analysis, summarizing the study results and exploring the limitations of the current research and recommendations for future work in this field.

While our systematic review showed a general trend for PVI to be higher in severe patients or non-survivors, it is evident that there are differing baseline results for the individual studies with 14 studies having reported significantly higher PVI (*p* ≤ 0.05). Furthermore, longitudinal analysis showed both increasing and decreasing trends during disease progression. PVI taken at the emergency room could be useful to guide decisions on immediate treatment. Additionally, monitoring PVI trends over time could be used to guide other aspects of therapy and to determine disease outcome. Nonetheless, we cannot markedly conclude that COVID-19 patients with elevated PVI are more likely to develop severe illness or are at higher risk of dying due to the variation in results.

Interestingly, some studies reported non-significant levels of PDW in severe patients but significantly higher MPV or P-LCR, either at admission or during the course of the disease (35, 42, 44). Studies evaluating the effect of storage time on platelet volume due to platelet swelling, identified a decrease in PDW and an increase in MPV (52, 53), indicating that PDW is a better indicator of platelet activation than MPV, since it was not elevated during single platelet distention caused by platelet swelling. It is possible that platelet swelling has occurred in these studies, though we can only surmise this has occurred due to absence of details on time to analysis and anticoagulant used.

Studies reporting that significantly higher PVI in severe or non-surviving patients was present at admission, also demonstrated that the platelet parameters were not independent predictors of disease progression (22, 47, 49), with age being the variable most likely to predict ICU requirement and mortality (23, 28). Patients with COVID-19 who enter a critical condition or die are mostly elderly, male and have comorbidities, with hypertension, diabetes and coronary artery disease being the most common (1, 54). PVI have been shown to be influenced by age, sex, hypertension, diabetes and coronary heart disease in some studies (55–60). Few included studies adjusted for confounding variables when assessing the association of PVI with COVID-19 disease, even though many reported significantly different ages and sex proportions when comparing the groups. Future studies should be designed to measure PVI in COVID-19 cohorts matching for age and gender. Giusti et al. (23) proposed a model, in which age and three routine coagulation parameters, including MPV, are measured to predict prognosis of hospitalized patients. Conceivably PVI could be used alongside other clinical and/or laboratory parameters to predict prognosis of COVID-19 disease rather than acting as a stand-alone biomarker.

A published letter has reported a significant association between MPV and combined severe illness and mortality in COVID-19 patients using pooled analysis of 18 studies (61). Pre-analytical and analytical variables were not considered however, and the statistical heterogeneity was extremely high (91%). Our review has identified many caveats in the current research which likely accounts for this heterogeneity including differences in participant characteristics, hematology analyzer, timing of the blood test, and clinical endpoints. As such we infer that the lack of robust standardization, along with the retrospective design and low patient numbers, renders the current research on PVI in COVID-19 patients inconclusive.

There is an inverse relationship between MPV and PLT in healthy adults (62). Increased MPV denotes an increase in circulating young platelets as a response to thrombocytopenia, possibly because of platelet consumption due to micro-thrombotic events in small vessels (63). Research has demonstrated that COVID-19 patients with thrombocytopenia have a significantly higher MPV and P-LCR, compared with COVID-19 patients with retained PLT (15). The general trend reported in our included studies showed a reduction in PLT at hospital admission, combined with increased PVI. Other studies have reported that patients with severe COVID-19 disease have a PLT only 23 × 10^9^/L to 31 × 10^9^/L lower than those with non-severe disease (64, 65). Bearing in mind that severely ill patients with systemic immune and coagulation activation maintain reasonable PLT, a compensatory platelet production response by the bone marrow at the early stage of the disease is probable. Notwithstanding, Barrett et al. (29) demonstrated that high MPV was independent of low PLT in COVID-19 patients. Therapies used early in the COVID-19 pandemic that cause thrombocytopenia, such as hydroxychloroquine and azithromycin, are given to COVID-19 patients at onset of disease (66, 67), possibly contributing to lower PLT in the later stages of the disease. Prospective studies that adjust for treatment could enable researchers to evaluate if thrombocytopenia is caused by disease progression and/or therapies.

It is important to note that research has shown platelet heterogeneity to be present from formation, and size may not necessarily reflect platelet age or activity. Platelet characteristics, including RNA content, may be dynamic over time (68–70). Thus, it is possible that platelet size heterogeneity predates COVID-19 infection. It also could reflect an inflammatory state due to other infection or co-morbidities. Therefore, the direction of effect cannot be inferred from the retrospective studies we review here, and the nature of this association must be elucidated by further prospective cause–effect analysis.

Research has shown that the median time for COVID-19 disease deterioration is 11 days to developing severe illness and entering a critical stage of severe pneumonia and organ damage (71). This corresponds with trends analysis by Kilercik et al. (24) who showed a distinctive divergence in MPV after 10 days when comparing non-survivors and survivors. Therefore, we propose that future work should comprise appropriately powered, prospective studies that analyze PVI trends over time, with consideration for confounders such as age, sex, co-morbidities and therapies. Notably, the majority of the included studies are from cohorts early in the pandemic before specific therapy was available, such as steroids, antivirals and vaccination, and with earlier COVID variants. Thus, the role of PVI biomarkers from early in the pandemic requires re-evaluation in the context of these factors with COVID-19 pneumonia now less common.

## Conclusion

While there is a trend toward higher PVI in severe COVID-19 patients and non-survivors, the contradictory findings of this systematic review suggest that further work is required to evaluate the potential usefulness of PVI for early prognosis of COVID-19. Most importantly, the technical concerns need to be addressed to fully demonstrate its use in clinical practice. Although some studies reported significant associations between clinical outcome and PVI, a causal relationship could not be inferred. Future studies should be prospective in design so that researchers can assess multiple outcomes at different time frames. They should give comprehensive methodology which includes careful study design, controlled measurement of platelet parameters, full reporting of how the data were acquired, and appropriate statistical considerations for confounding factors.

## Data availability statement

The raw data supporting the conclusions of this article will be made available by the authors, without undue reservation.

## Author contributions

SD made substantial contributions to the design of the study, with support from HW, DD, and MT. SD and HW carried out the full search and screened the literature for inclusion and exclusion and performed the data extraction and quality assessment. SD performed the analysis and interpretation of the data and drafted the first version. Substantial revisions were provided by HW, DD, and MT. All authors read and approved the final version of the manuscript.

## Funding

This review was supported by the UK Research and Innovation (UKRI) and National Institute for Health and Care Research (NIHR), Grant Ref: MC_PC_19083. The funder did not contribute to the study design, analysis or writing of the report.

## Conflict of interest

The authors declare that the research was conducted in the absence of any commercial or financial relationships that could be construed as a potential conflict of interest.

## Publisher's note

All claims expressed in this article are solely those of the authors and do not necessarily represent those of their affiliated organizations, or those of the publisher, the editors and the reviewers. Any product that may be evaluated in this article, or claim that may be made by its manufacturer, is not guaranteed or endorsed by the publisher.

## Abbreviations

CBC: Complete Blood Count
CENTRAL: Cochrane Central Register of Controlled Trials
CNKI: China Knowledge Resource Integrated
COVID-19: Coronavirus disease 2019
EDTA: Ethylenediaminetetraacetic acid
fL: Femtoliters
ICU: Intensive care unit
IQR: Interquartile range
LMR: Lymphocyte/mean platelet volume ratio
MPV: Mean platelet volume
MPR: Mean platelet volume/platelet count ratio
PDW: Platelet distribution width
P-LCR: Platelet-large cell ratio
PLT: Platelet count
PRISMA: Preferred Reporting Items for Systematic Reviews and Meta-Analyses
PVI: Platelet volume indices
SARS-CoV-2: Severe acute respiratory syndrome coronavirus 2.
