# Supplementary material for: Are platelet volume indices of clinical use in COVID-19? A systematic review
Source: Front Cardiovasc Med. 2022 Oct 18;9:1031092. doi: 10.3389/fcvm.2022.1031092 (PMC9623063; doi:10.3389/fcvm.2022.1031092)
Supplement: Supplementary file 1 [file Table_1.DOCX]

**Search Strategy**

Medline

1. (mean platelet volume* or platelet distribution width* or platelet large cell ratio*).mp.

2. limit 1 to english language

3. (covid or coronavirus or ncov or sars or sars-cov*).mp.

4. limit 3 to english language

5. 2 and 4
